# Heart Rhythm Complexity Impairment in Patients with Pulmonary Hypertension

**DOI:** 10.1038/s41598-019-47144-1

**Published:** 2019-07-24

**Authors:** Cheng-Hsuan Tsai, Hsi-Pin Ma, Yen-Tin Lin, Chi-Sheng Hung, Mi-Chia Hsieh, Ting-Yu Chang, Ping-Hung Kuo, Chen Lin, Men-Tzung Lo, Hsao-Hsun Hsu, Chung-Kang Peng, Yen-Hung Lin

**Affiliations:** 1 Department of Internal Medicine, National Taiwan University Hospital and National Taiwan University College of Medicine, Taipei, Taiwan; 2 Department of Electrical Engineering, National Tsing Hua University, Hsinchu, Taiwan; 3 Department of Internal Medicine, Taoyuan General Hospital, Taoyuan, Taiwan; 4 Department of Biomedical Sciences and Engineering, National Central University, Chungli, Taiwan; 5 Department of Surgery, National Taiwan University Hospital and National Taiwan University College of Medicine, Taipei, Taiwan; 6 Division of Interdisciplinary Medicine and Biotechnology, Beth Israel Deaconess Medical Center/Harvard Medical School, Boston, Massachusetts, USA

**Keywords:** Applied mathematics, Scientific data, Cardiovascular diseases, Electrodiagnosis, Diagnostic markers

## Abstract

Pulmonary hypertension is a fatal disease, however reliable prognostic tools are lacking. Heart rhythm complexity analysis is derived from non-linear heart rate variability (HRV) analysis and has shown excellent performance in predicting clinical outcomes in several cardiovascular diseases. However, heart rhythm complexity has not previously been studied in pulmonary hypertension patients. We prospectively analyzed 57 patients with pulmonary hypertension (31 with pulmonary arterial hypertension and 26 with chronic thromboembolic pulmonary hypertension) and compared them to 57 age- and sex-matched control subjects. Heart rhythm complexity including detrended fluctuation analysis (DFA) and multiscale entropy (MSE) and linear HRV parameters were analyzed. The patients with pulmonary hypertension had significantly lower mean RR, SDRR, pNN_20_, VLF, LF, LF/HF ratio, DFAα1, MSE slope 5, scale 5, area 1–5 and area 6–20 compared to the controls. Receiver operating characteristic curve analysis showed that heart rhythm complexity parameters were better than traditional HRV parameters to predict pulmonary hypertension. Among all parameters, scale 5 had the greatest power to differentiate the pulmonary hypertension patients from controls (AUC: 0.845, *P* < 0.001). Furthermore, adding heart rhythm complexity parameters significantly improved the discriminatory power of the traditional HRV parameters in both net reclassification improvement and integrated discrimination improvement models. In conclusion, the patients with pulmonary hypertension had worse heart rhythm complexity. MSE parameters, especially scale 5, had excellent single discriminatory power to predict whether or not patients had pulmonary hypertension.

## Introduction

Pulmonary hypertension is a progressive and debilitating diseases caused by complex and heterogeneous pathogeneses^1,2^. Pulmonary artery hypertension (PAH) and chronic thromboembolic pulmonary hypertension (CTEPH) are important subgroups of pulmonary hypertension which share similar hemodynamic physiology^3,4^. Both diseases are characterized by progressive precapillary vessel arteriopathy with progressive elevated pulmonary vascular resistance^5,6^, and patients with these diseases have a poor prognosis if untreated^7,8^. The major causes of death include right heart failure, sudden cardiac death and respiratory failure^9,10^.

Changes in linear heart rate variability (HRV) have been reported to be associated with pulmonary hypertension and its cardiovascular outcomes^11–13^. In addition, changes in linear HRV have been shown to be partially reversible after treatment with subcutaneous treprostinil^14^. This suggests that the deterioration in linear HRV in patients with pulmonary hypertension may be associated with hemodynamic abnormalities.

In addition to traditional linear HRV parameters, new methods using non-linear HRV analysis have been developed in recent years which have focused on measuring complexity instead of variability beneath heart rate dynamics^15,16^. Two of the most frequently used methods to estimate heart rhythm complexity are detrended fluctuation analysis (DFA) and multiscale entropy (MSE). In previous studies, both DFA and MSE have shown better predictive ability for clinical outcomes in many diseases compared with traditional HRV analysis^17–20^. However, studies of heart rhythm complexity in patients with pulmonary hypertension are lacking. In this study, we aimed to evaluate changes in heart rhythm complexity in patients with pulmonary hypertension and the potential clinical applications.

## Results

### Patient characteristics

The clinical, echocardiographic and hemodynamic data and information on pulmonary hypertension-specific medications are presented in Table 1. The patients with pulmonary hypertension had a significantly lower body mass index (BMI), lower prevalence of hypertension and higher tricuspid regurgitation peak gradient (TRPG) than the controls. In medication, the control group had significantly higher rates of beta blocker, calcium channel blocker (CCB) and angiotensin II receptor blocker (ARB) or angiotensin-converting enzyme inhibitors (ACEI) use. Other parameters were compatible between the two groups except for data on hemodynamics and medications for pulmonary hypertension which were only available in the patients with pulmonary hypertension. The mean pulmonary arterial pressure, pulmonary capillary wedge pressure, cardiac output and pulmonary vascular resistance in the patients with pulmonary hypertension were 46 ± 15 mmHg, 13 ± 4 mmHg, 4.1 ± 1.5 L/min and 723 ± 419 dyn·s·cm^−5^, respectively.

**Table 1:** Clinical data of the patients.

	Pulmonary hypertension (N = 57)	Control (N = 57)	P Value
Age, years	55 ± 16	57 ± 10	0.305
Male, n(%)	23 (40%)	31 (54%)	0.133
BMI	24 ± 4	26 ± 4	0.011
CAD, n(%)	3 (5%)	1 (2%)	0.309
DM, n(%)	6 (11%)	8 (14%)	0.568
HTN, n(%)	11 (19%)	27 (47%)	0.001
**Medication**			
Beta blocker, n(%)	4 (7%)	28 (49%)	<0.001
ACEI or ARB, n(%)	5 (9%)	12 (21%)	<0.001
CCB, n(%)	10 (18%)	22 (39%)	0.012
**Biochemistry**			
Glucose AC, mg/dL	105 ± 23	98 ± 15	0.110
Creatinine, mg/dL	1.0 ± 0.5	0.9 ± 0.2	0.329
TG, mg/dL	109 ± 57	117 ± 53	0.525
T -Chol, mg/dL	164 ± 49	179 ± 38	0.125
**Echocardiogram**			
LVEF, %	68 ± 15	70 ± 6.0	0.240
TRPG, mmHg	73 ± 30	23 ± 5	<0.001
**Hemodynamic data**			
Mean PAP, mmHg	46 ± 15	—	—
PCWP, mmHg	13 ± 4	—	—
Cardiac output, L/min	4.1 ± 1.5	—	—
PVR, dyn·s·cm^−5^	723 ± 419	—	—
PAH specific medication		—	—
sildenafil, n(%)	24 (42%)	—	—
macitentan, n(%)	4 (7%)	—	—
riociguat, n(%)	7 (12%)	—	—
bosentan, n(%)	2 (4%)	—	—
iloprost, n(%)	1 (2%)	—	—
treprostinil, n(%)	1 (2%)	—	—

Data were presented as mean ± standard deviation or number (percentage).

Abbreviation: BMI = body mass index; CAD = coronary artery disease; DM = diabetes mellitus; HTN = hypertension; ACEI = angiotensin-converting enzyme inhibitors; ARB = angiotensin receptor blockers; CCB = calcium channel blocker; TG = triglyceride; T-Chol = total cholesterol; LVEF = left ventricular ejection fraction; TRPG = tricuspid regurgitation peak gradient; PAP = pulmonary arterial pressure; PCWP = pulmonary capillary wedge pressure; PVR = pulmonary vascular resistance; PGE1 = prostaglandin E1.

### Holter data

**Figure 1:**
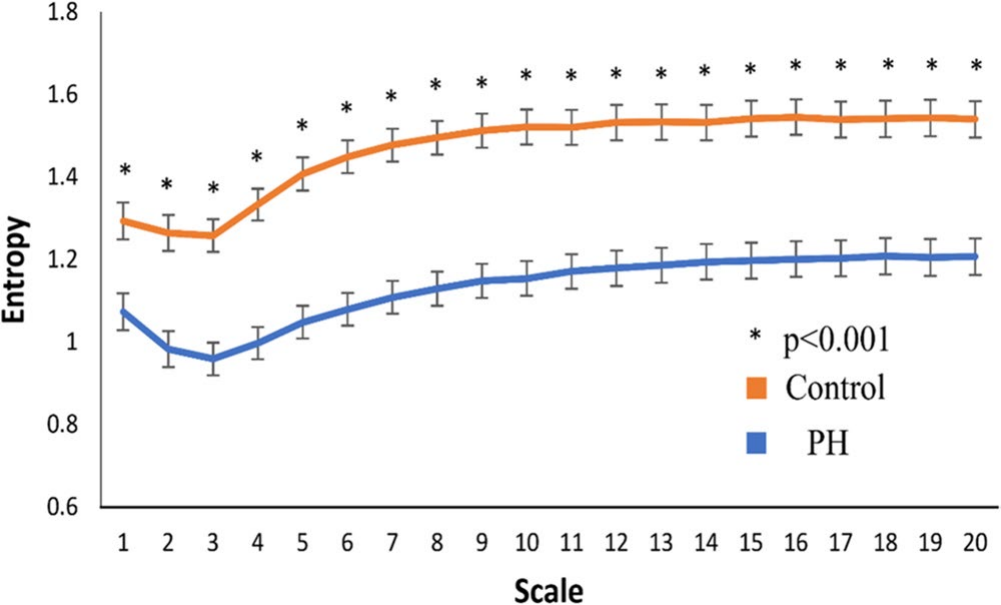
The entropy over different time scales in patients with (blue) and without (orange) pulmonary hypertension. *p < 0.001.

In linear analysis, the patients with pulmonary hypertension had significantly lower mean RR, standard deviation of R-R intervals (SDRR), percentage of absolute differences in normal RR intervals greater than 20 ms (pNN_20_), very low frequency (VLF), low frequency (LF) and low frequency/high frequency (LF/HF) ratio compared to the controls (Table 2). In heart rhythm complexity parameters including MSE and DFA, DFAα1, MSE slope 5, scale 5, area under the MSE curve for scale 1–5 (area 1–5) and 6–20 (area 6–20) were significantly lower in the pulmonary hypertension group compared to the control group. The value of DFAα2 was comparable between the two groups (Table 2). The entropies of different time scales of MSE curves were significantly different between the patients with and without pulmonary hypertension (Fig. 1).

**Table 2:** Holter Parameters in patients with pulmonary hypertension and control.

	Pulmonary hypertension (N = 57)	Control (N = 57)	P Value
**Time Domain Analysis**			
Mean RR, ms	756.150 (681.840~841.155)	832.450 (718.585~887.660)	0.003
SDRR, ms	64.862 (53.032~81.562)	73.578 (64.042~87.078)	0.043
pNN_20_, %	0.249 (0.149~0.356)	0.314 (0.199~0.399)	0.016
pNN_50_, %	0.035 (0.009~0.071)	0.027 (0.011~0.062)	0.899
**Frequency Domain Analysis**			
VLF, ms^2^	303.870 (157.780~529.665)	445.050 (337.525~664.040)	0.001
LF, ms^2^	87.675 (40.313~172.665)	122.490 (75.639~195.215)	0.045
HF, ms^2^	55.431 (18.293~147.955)	33.234 (24.493~73.112)	0.214
LF/HF ratio	1.235 (0.887~2.735)	3.292 (1.877~4.391)	<0.001
**Detrended fluctuation analysis**			
DFAα1	0.963 (0.795~1.147)	1.262 (1.044~1.331)	<0.001
DFAα2	1.109 (1.038~1.172)	1.122 (1.070~1.154)	0.411
**Multiscale entropy**			
Slope 5	0.003 (−0.054~0.060)	0.046 (−0.008~0.077)	0.008
Scale 5	1.054 (0.862~1.234)	1.436 (1.247~1.557)	<0.001
Area 1–5	4.183 (3.116~4.772)	5.155 (4.355~5.651)	<0.001
Area 6–20	16.872 (14.003~19.771)	21.216 (18.912~22.756)	<0.001

Data were presented as Values are median (25^th^–75^th^ percentile). Abbreviation: SDRR = standard deviation of normal RR intervals; pNN_20_ = percentage of the absolute change in consecutive normal RR interval exceeds 20 ms; pNN_50_ = percentage of the absolute change in consecutive normal RR interval exceeds 50 ms; VLF = very low frequency; LF: low frequency; HF = high frequency; DFA = detrended fluctuation analysis.

### Logistic regression analysis to predict pulmonary hypertension

In univariate logistic regression, lower linear HRV parameters including mean RR, VLF and LF/HF ratio, and lower heart rhythm complexity including DFAα1, MSE slope 5, scale 5, area 1–5 and area 6–20 were significantly associated with the presence of pulmonary hypertension. These parameters were further analyzed using multivariate logistic regression, which showed that lower mean RR, DFAα1 and scale 5 were significantly associated with pulmonary hypertension (Table 3). Then, these 3 parameters including mean RR, DFAα1 and MSE scale 5 were adjusted by age, sex, BMI, HTN, DM, beta blocker, CCB and ARB or ACEI use in different models. In the five models with different adjustments, only DFAα1 and MSE scale 5 remained as independent predictors of pulmonary hypertension (Table 4).

**Table 3:** Univariate and multivariate logistic regression model to predict the presence of pulmonary hypertension.

Univariate logistic regression			Multivariate logistic regression	
	β (95% C.I)	P	OR (95% C.I)	P
Mean RR	0.995 (0.991~0.998)	0.004	0.994 (0.989~0.999)	0.017
SDRR	0.987 (0.970~1.004)	0.121		
pNN_20_	0.097 (0.09~1.037)	0.054		
pNN_50_	6.334 (0.122~329.362)	0.360		
VLF	0.998 (0.997~0.999)	0.008		
LF	1.000 (0.998~1.001)	0.942		
HF	1.003 (0.999~1.006)	0.138		
LF/HF ratio	0.691 (0.544~0.877)	0.002		
DFAα1	0.022 (0.004~0.139)	<0.001	0.022 (0.002~0.200)	0.001
DFAα2	0.127 (0.003~5.870)	0.291		
Slope 5	<0.001 (<0.001~0.087)	0.007		
Scale 5	0.003 (<0.001~0.028)	<0.001	0.004 (<0.001~0.037)	<0.001
Area 1–5	0.356 (0.223~0.566)	<0.001		
Area 6–20	0.697 (0.599~0.810)	<0.001		

*In multivariate logistic regression, the VLF, LF/HF ratio, slope 5, area 1–5 and area 6–20 were excluded from the model.

Abbreviation: SDRR = standard deviation of normal RR intervals; pNN_20_ = percentage of the absolute change in consecutive normal RR interval exceeds 20 ms; pNN_50_ = percentage of the absolute change in consecutive normal RR interval exceeds 50 ms; VLF = very low frequency; LF = low frequency; HF = high frequency; DFA = detrended fluctuation analysis.

**Table 4:** Heart rhythm complexity to predict pulmonary hypertension after adjustment.

	Mean RR*		DFAα1*		Scale5*	
	β (95% C.I)	P value	β (95% C.I)	P value	β (95% C.I)	P value
Model 1	0.994 (0.989~0.999)	0.017	0.022 (0.002~0.200)	0.001	0.004 (<0.001~0.037)	<0.001
Model 2	0.995 (0.990~1.000)	0.070	0.013 (0.001~0.145)	<0.001	0.002 (<0.001~0.023)	<0.001
Model 3	0.996 (0.991~1.001)	0.126	0.009 (0.001~0.110)	<0.001	0.002 (<0.001~0.025)	<0.001
Model 4	0.995 (0.989~1.000)	0.070	0.007 (<0.001~0.102)	<0.001	0.001 (<0.001~0.014)	<0.001
Model 5	0.995 (0.989~1.001)	0.103	0.005 (<0.001~0.088)	<0.001	0.001 (<0.001~0.021)	<0.001

Model 1 unadjusted.

Model 2 adjusted by age and sex.

Model 3 adjusted by age, sex, BMI.

Model 4 adjusted by age, sex, BMI, HTN and DM.

Model 5 adjusted by age, sex, BMI, HTN, DM, beta blocker, CCB and ARB or ACEI use.

*Independent predictors of pulmonary hypertension in multivariate logistic regression model including mean RR, VLF, LF/HF ratio, DFAα1, slope 5, scale 5, area 1–5 and area 6–20 after stepwise subset selection.

Abbreviation: DFA = detrended fluctuation analysis.

### Comparisons of all linear HRV and heart rhythm complexity parameters to differentiate the patients with and without pulmonary hypertension

Receiver operating characteristic (ROC) curve analysis showed that MSE scale 5 had the greatest discriminatory power to predict the presence of pulmonary hypertension among all linear and heart rhythm complexity parameters (area under the curve [AUC] 0.845).

**Figure 2:**
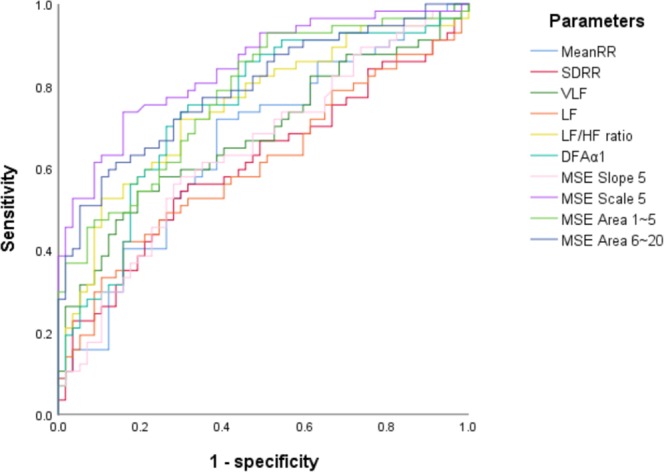
Analysis of the discrimination power of the two group by receiver operating characteristic curve analysis. The areas under the curve of mean RR, SDRR, VLF, LF, LF/HF ratio, DFAα1, MSE slope 5, scale 5, area 1–5 and area 6–20 were 0.660, 0.610, 0.681, 0.609, 0.748, 0.745, 0.644, 0.845, 0.777 and 0.794, respectively.

The AUCs of other linear and non-linear HRV parameters including mean RR, SDRR, pNN_20_, pNN_50_, VLF, LF, HF, LF/HF ratio, DFAα1, DFAα2, slope 5, area 1–5, and area 6–20 were 0.660, 0.610, 0.630, 0.493, 0.681, 0.609, 0.432, 0.748, 0.745, 0.545, 0.644, 0.777 and 0.794, respectively (Fig. 2). In addition, the AUCs of clinical parameters including BMI, coronary artery disease, diabetes mellitus and hypertension were 0.355, 0.526, 0.489 and 0.341, respectively.

### The advantage of adding DFA or MSE parameters to the linear parameters to discriminate the presence of pulmonary hypertension

MSE parameters including scale 5, area 1–5 and area 6–20 significantly improved the discriminatory power of mean RR, pNN_20_, VLF, LF and LF/HF ratio in both net reclassification improvement (NRI) and integrated discrimination improvement (IDI) models. DFAα1 significantly improved the discriminatory power of mean RR, pNN_20_, VLF and LF in both NRI and IDI models and LF/HF ratio in IDI model (Table 5).

**Table 5:** AUC, NRI, and IDI models of linear parameters before and after adding DFAα1 and MSE parameters to discriminate patients with or without pulmonary hypertension.

Parameters		AUC	R square	NRI	NRI p-value	IDI	IDI p-value
Mean RR		0.66	0.08				
	+DFAα1	0.781	0.232	0.877	<0.001	0.16	<0.001
	+Scale5	0.857	0.354	1.018	<0.001	0.311	<0.001
	+Area1-5	0.793	0.229	0.596	0.001	0.162	<0.001
	+Area6-20	0.813	0.264	0.877	<0.001	0.223	<0.001
pNN_20_		0.63	0.034				
	+DFAα1	0.821	0.269	0.982	<0.001	0.257	<0.001
	+Scale5	0.844	0.33	0.947	<0.001	0.319	<0.001
	+Area1-5	0.772	0.216	0.667	<0.001	0.189	<0.001
	+Area6-20	0.801	0.253	0.842	<0.001	0.246	<0.001
VLF		0.681	0.068				
	+DFAα1	0.768	0.199	0.772	<0.001	0.139	<0.001
	+Scale5	0.851	0.336	0.947	<0.001	0.294	<0.001
	+Area1-5	0.8	0.231	0.526	0.003	0.182	<0.001
	+Area6-20	0.799	0.259	0.807	<0.001	0.212	<0.001
LF		0.609	<0.001				
	+DFAα1	0.75	0.175	0.877	<0.001	0.182	<0.001
	+Scale5	0.852	0.353	1.123	<0.001	0.376	<0.001
	+Area1-5	0.789	0.251	0.702	<0.001	0.254	<0.001
	+Area6-20	0.797	0.248	0.807	<0.001	0.274	<0.001
LF/HF ratio		0.748	0.088				
	+DFAα1	0.751	0.174	0.211	0.255	0.067	0.006
	+Scale5	0.861	0.365	0.982	<0.001	0.279	<0.001
	+Area1-5	0.82	0.284	0.702	<0.001	0.188	<0.001
	+Area6-20	0.806	0.266	0.807	<0.001	0.177	<0.001

pNN_20_ = percentage of the absolute change in consecutive normal RR interval exceeds 20 ms; VLF = very low frequency; LF = low frequency; HF = high frequency; AUC = areas under the curve; NRI = net reclassification improvement; IDI = integrated discrimination improvement; MSE = multiscale entropy; DFA = detrended fluctuation analysis.

## Discussion

The three major findings of this study are: (1) the patients with pulmonary hypertension had both worse HRV and heart rhythm complexity compared to those without pulmonary hypertension; (2) MSE scale 5 had the greatest single discriminatory power to detect the presence of pulmonary hypertension among all HRV and clinical parameters; (3) the combination of linear HRV and heart rhythm complexity parameters improved the discriminatory power to predict pulmonary hypertension.

Patients with pulmonary hypertension have a poor prognosis, even after using pulmonary hypertension-specific drugs^21^. The major causes of death are right heart failure and sudden death, which account for about 60% of all cases of mortality^22,23^. Unlike in left heart failure, ventricular tachycardia or fibrillation is relatively rare in patients with pulmonary hypertension. Instead, severe bradycardia and pulseless electrical activity are the most common causes of sudden cardiac death in PAH^22^. A possible predisposing factor for arrhythmia in PAH is modulation of autonomic activity^9,22,24^. Elevated levels of serum norepinephrine and its association with pulmonary vascular resistance^25^ support the hypothesis of increased sympathetic activity in patients with PAH.

Heart rate variability is a validated and non-invasive tool to evaluate cardiac autonomic function^26^. Folino *et al*. reported decreased HRV and increased ventricular ectopy in patients with PAH^27^, and Bienias and Witte *et al*. also reported worse linear HRV in patients with pulmonary hypertension^23,24^. In the current study, we demonstrated similar results which highlight the prominent autonomic dysregulation in patients with pulmonary hypertension. In addition to linear HRV analysis, heart rhythm complexity analysis derived from non-linear HRV analysis has been studied as a better predictor of outcomes in many diseases compared with linear analysis. Data obtained from the DIAMOND-CHF trial showed that heart rhythm complexity impairment was the strongest electrocardiographic risk predictor, exceeding the value of traditional linear HRV analysis^28^. However, non-linear HRV analysis has never been reported in patients with pulmonary hypertension. To the best of our knowledge, non-linear HRV analysis has only been reported in one animal model experiment, in which Gonçalves *et al*. demonstrated decreases in both linear and non-linear HRV parameters in a rat model of monocrotaline-induced pulmonary hypertension^29^.

Heart rhythm complexity derived from non-linear analysis including DFA and MSE based on fractal and chaos theories, respectively, focuses on measuring the complexity beneath seemingly stationary biological signals^15,16^. A normal healthy subject is capable of making adjustments to deal with a dynamic environment through highly complex multisystemic cooperation. In a diseased subject, the balance in the systems breaks down and the complexity decreases. Heart rhythm complexity analysis can quantify this complexity, and it has been studied in many different diseases with excellent results. It has been associated with the prognosis of heart failure^20^, outcomes of acute stroke^17^, primary aldosteronism^30^, severity of abdominal aorta calcification^19^, critical illnesses requiring extracorporeal life support^18^ and post-myocardial infarction heart function^31^. In the current study, heart rhythm complexity, and especially MSE scale 5, had a better discriminatory power for pulmonary hypertension compared to linear HRV analysis. The DFAα1 and MSE scale 5 remained as independent predictors of pulmonary hypertension after clinical parameters adjustments. Furthermore, a combination of heart rhythm complexity and linear HRV analysis further significantly improved the predictive power of linear HRV parameters to differentiate between the patients with and without pulmonary hypertension. Our results provide valuable evidence supporting an altered autonomic system and decreased heart rhythm complexity in patients with pulmonary hypertension.

There are several limitations to this study. First, this is a small pilot study and the data were only derived from both PAH and CTEPH patients. Patients with other pulmonary hypertension groups such as group 2 (left heart disease related) or group 3 (pulmonary disease/hypoxia related) were not included in this study. The results of this study should be confirmed in larger clinical studies. Second, the baseline characteristics including BMI, prevalence of HTN and medication were different in control and pulmonary hypertension groups which may still be confounders in this study. Third, the baseline physical activity difference between these two group may influence the HRV parameters and may also be a confounder in this study. Fourth, this is a cross-sectional study without long-term follow-up data. Further studies are needed to evaluate the prognostic value of heart rhythm complexity in patients with pulmonary hypertension.

In conclusion, heart rhythm complexity could predict the presence of pulmonary hypertension in this study, and MSE scale 5 had the greatest single discriminatory power. In addition, heart rhythm complexity parameters including DFA and MSE significantly improved the discriminatory power of linear HRV parameters, which supports the advantage of combining linear and heart rhythm complexity parameters.

## Methods

### Patients

We prospectively enrolled 57 Taiwanese patients with pulmonary hypertension, including 31 patients with PAH (World Health Organization, WHO group 1) and 26 patients with CTEPH (WHO group 4). Patients with left heart disease (WHO group 2) and COPD (WHO group 3) were not enrolled in this study to prevent bias deprived from complexed and heterogenous disease mechanisms among different pulmonary hypertension groups. The diagnosed and categorized of pulmonary hypertension were based on ESC guideline. For the control group, we enrolled 57 age- and sex-matched participants who admitted to our hospital and received coronary angiogram examination which revealed patent coronary artery. Patients with chronic pulmonary disease, chronic atrial fibrillation, prior myocardial infarction, left heart failure, cerebrovascular events, or peripheral artery disease were excluded.

All subjects in this study received echocardiography and 24-h ambulatory ECG Holter recording. All patients with pulmonary hypertension received right heart catheterization to confirm the diagnosis. The baseline characteristics, medical history and biochemistry data were recorded at enrollment. Holter recordings were performed one month before or after (mostly one week before) right heart catheterization in the patients with pulmonary hypertension. In the control group, Holter recordings were performed within one week after coronary angiogram.

This study was approved by the Institutional Review Board of National Taiwan University Hospital, and all subjects provided written informed consent. All research was performed in accordance with relevant guidelines and regulations.

### Echocardiography

All subjects received standard transthoracic echocardiography (iE33 xMATRIX Echocardiography System, Philips, Amsterdam, Netherlands). The TRPG was determined from the peak flow velocity of tricuspid regurgitation (TRV) using a simplified Bernoulli equation: TRPG = 4 × TRV^2^, and LVEF (M-mode) was measured via a parasternal long axis view in accordance with the recommendations of the American Society of Echocardiography^32^.

### 24-hour Holter recording and data pre-processing

24-h ambulatory ECG Holter (Zymed DigiTrak Plus 24-Hour Holter Monitor Recorder and Digitrak XT Holter Recorder 24 Hour, Philips, Amsterdam, Netherlands) recordings were conducted in all subjects. All subjects maintained their daily activity during the examination. A stable 4-h segment of daytime RR intervals (between 9 AM and 5 PM) was selected. The selected data were automatically annotated using an algorithm, and then examined by two experienced technicians. MATLAB program with self-writing code was used to derive HRV parameters for signal processing.

### Linear analysis

Traditional linear HRV including time and frequency domain analysis was conducted according to the recommendations of the North American Society of Pacing Electrophysiology and the European Society of Cardiology^33^. Time domain HRV parameters including mean RR, SDRR, pNN_20_ and pNN_50_ were calculated to represent the sympathetic and parasympathetic modulation of heart beats. The frequency domain parameters, high frequency (HF; 0.15–0.4 Hz), low frequency (LF; 0.04–0.15 Hz), and very low frequency (VLF; 0.003–0.04 Hz) were analyzed after Fourier transformation.

### Non-linear analysis

Non-linear HRV analysis focuses on the complexity of heart rate dynamics. Two non-linear methods, MSE and DFA, were conducted in this study based on fractal and chaos theories, respectively.

### Detrended fluctuation analysis

DFA provides a mathematical algorithm to uncover the fractal behavior beneath seemingly nonstationary RR dynamics by removing these trends from the integrated time series and quantifying the degree of self-affinity based on fractal theory^15^. DFA was performed by summing the detrended integrated time series in each scale. The log-log plots of fluctuations against time scales were constructed, and the slope (α exponent) of the plot represented the fractal correlation property of the time series. A crossover phenomenon of α exponents of RR dynamics was observed in normal and diseased subjects. Therefore, short (α1; 4–11 beats) and long (α2; 11–64 beats) time scales were calculated to better understand the fractal property of the heart rate dynamics.

### Multiscale entropy analysis

MSE analysis can be used to estimate the entropies of physiological signals in different time scales, and it can also be used to predict sequential changes over different time scales^16^. Since traditional entropy analysis can only evaluate the single entropy of a biological signal, MSE uses a coarse-graining process (i.e. averaging consecutive beats to form a new time series) to construct many different time scales. After this process, the estimated entropies over different time scales can be calculated, which represent the complexity of the physiological signals^34^. In this study, the entropy values of scale 5 (scale 5), the linear-fitted slope of scale 1–5 (slope 5), the summation of entropy values of scales 1–5 (area 1–5) and 6–20 (area 6–20) were calculated as MSE parameters to quantify the complexity of the RR dynamics exhibited in short and long time scales.

### Statistical analysis

Data were expressed as mean ± standard deviation and median (25^th^ and 75^th^ percentiles) for normally distributed and non-normally distributed data, respectively. Comparisons of data between patients with and without pulmonary hypertension were made using the independent t-test and the Mann-Whitney U test. Differences between proportions were assessed using the chi-square test or Fisher’s exact test. Logistic regression analysis was used to validate associations between parameters and the presence of pulmonary hypertension. Significant determinants in univariate logistic regression analysis (P < 0.05) including mean RR, VLF, LF/HF ratio, DFAα1, slope 5, scale 5, area 1–5 and area 6–20 were then tested in multivariate logistic regression analysis with stepwise subset selection to identify independent factors to predict the presence of pulmonary hypertension. Then the independent HRV predictors of pulmonary hypertension in the multivariate regression model including mean RR, DFAα1 and MSE scale 5 were adjusted by clinical parameters including age, sex, BMI, HTN, DM, beta blocker, CCB and ARB or ACEI use in 5 logistic regression models. The AUC was used to assess the discriminatory power of the model to predict pulmonary hypertension. Furthermore, NRI and IDI were used to evaluate improvements in the predictive power after adding a single heart rhythm complexity parameter into a logistic regression model using only linear parameters^35^. The significance of NRI and IDI statistics was based on approximate normal distributions. All statistical analyses were performed using R software (http://www.r-project.org/) and SPSS version 25 for Windows (SPSS Inc., IL, USA). The significance level of the statistical analysis was set at 0.05.
